# Towards Qualification of Epoxy Resins for Superconducting Magnets Exposed to Radiation Doses Exceeding 100 MGy: Effect of the Radiation Source and Environment

**DOI:** 10.3390/polym18091079

**Published:** 2026-04-29

**Authors:** Christian Scheuerlein, Federico Ravotti, Giuseppe Pezzulo, Torsten Koettig, Oliver Aberle, Ana-Paula Bernardes, Roland Piccin, Michael Eisterer

**Affiliations:** 1 European Organization for Nuclear Research (CERN), Esplanade des Particules 1, 1211 Geneva, Switzerland; 2 Atominstitut, TU Wien, Stadionallee 2, 1020 Vienna, Austria

**Keywords:** epoxy resin, total absorbed dose, gamma irradiation, proton irradiation, neutron irradiation, DMA, chain scission, cross-linking, superconducting magnet, HL-LHC, FCC, muon collider

## Abstract

To qualify epoxy resin systems for use in superconducting magnets of future particle accelerators up to peak doses beyond 100 MGy, the effects of the irradiation source, the irradiation environment and the irradiation temperature have been assessed. Identical epoxy resin samples have been irradiated with ^60^Co gamma rays, 24 GeV/c protons and by mixed neutron/gamma radiation in a reactor and at a spallation source up to a dose of 170 MGy. Irradiation-induced cross-linking and chain scission have been monitored by Dynamical Mechanical Analysis (DMA). When irradiations are performed with the same dose rate and in the same environment, the different radiation sources have a similar efficiency to produce radiation damage, and the total absorbed dose is a good scaling factor to compare irradiation effects in polymers. To distinguish between the influence of the irradiation temperature and of environmental oxygen, proton irradiations have been carried out in ambient air, inert gas at ambient temperature and in liquid helium. Compared to ambient air irradiation, in inert atmosphere more cross-linking is observed. Cross-linking rates are strongly reduced at 4.2 K. For some polymers the irradiation temperature has a strong influence on the chain scission rate. The most-radiation-hard epoxy resin systems maintain substantial mechanical strength up to doses beyond 100 MGy.

## 1. Introduction

**Figure 1:**
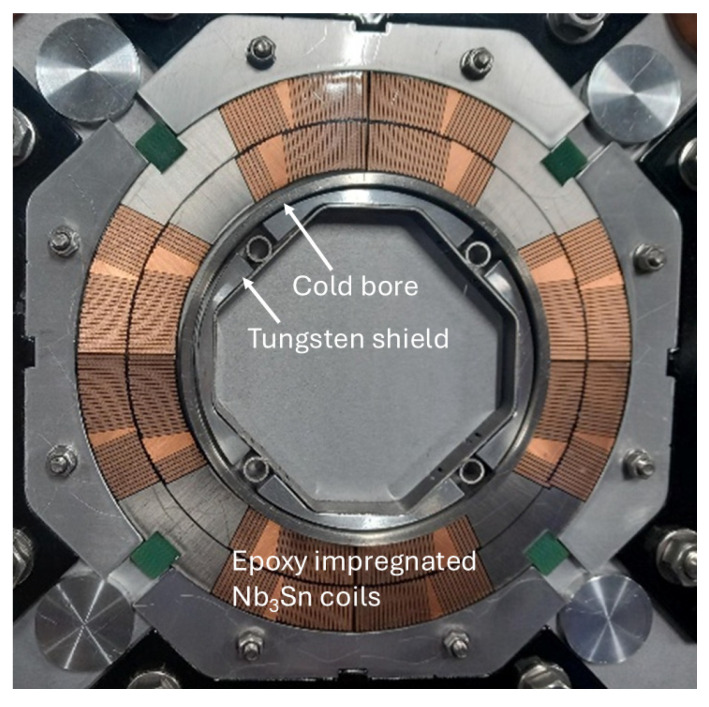
MQXFB magnet cross section with tungsten shielding inside the magnet cold bore.

Superconducting magnets of future particle accelerators like the High Luminosity Large Hadron Collider (HL-LHC) [1,2], the Future Circular Collider (FCC-hh) [3,4] and a future Muon Collider (MuCol) [5,6] will be exposed to increasingly high radiation doses. For example, in the HL-LHC final focus magnets, 0.6–1.6 cm thick internal tungsten shielding is required to limit the peak dose to the organic insulation materials to 30 MGy, which is assumed to be the safe, conservative dose limit [7,8]. The shielding reduces the effective magnet bore diameter by 1.2–3.2 cm (Figure 1).

To keep the radiation dose in the FCC-hh final focus magnet coils below 80 MGy, an internal tungsten shielding thickness of 4 cm is required [4,9]. A more precise knowledge of the dose limits of the insulation constituents is needed to further optimise shielding design and improve magnet performance.

An increase in the dose limits of organic insulation systems can come from the choice of the most-radiation-hard insulation materials, a better understanding of the effect of different irradiation sources, irradiation environments and irradiation temperature, and by refining dielectric and mechanical requirements of superconducting magnet insulation systems. The present paper compares the effect of different irradiation sources, the irradiation environment and temperature.

Extensive polymer radiation-damage test programmes have been carried out for decades [10]. As an example, at CERN, radiation damage of organic materials used in particle accelerators and in their environment has been systematically studied [11,12,13]. An important irradiation test programme has also been performed for the qualification of the insulation system of the ITER Toroidal Field (TF) coils [14,15,16].

For the above-mentioned studies, mainly two types of irradiation sources have been used. Irradiations to lower dose levels have been done using ^60^Co gamma irradiation in ambient air with dose rates of the order of 1 kGy/h. Very high doses are typically achieved by mixed neutron/gamma irradiation in fission reactors, where dose rates of the order of 1 MGy/h can be obtained. Irradiation damage assessment of pure polymers and fibre-reinforced composites has been based on the relative changes of a critical property, for instance, the flexural strength, the deformation at break, or the shear strength [17,18,19].

In the present study, we compare the ageing of different epoxy resin systems when exposed to different radiation sources, notably mixed neutron/gamma radiation from a spallation source, mixed gamma/neutron irradiation from a fission reactor, ^60^Co gamma rays and 24 GeV/c protons, with dose rates varying by three orders of magnitude.

To compare the efficiency of the different sources for producing radiation damage, radiation effects like cross-linking and chain scission to doses up to 170 MGy are monitored by measurements of the glass transition temperature (*T*_g_) and of the rubbery shear modulus (*G*′_rubbery_) by Dynamic Mechanical Analysis (DMA) [20] and by complementary three-point bending tests with identical materials. The effect of the radiation environment has been studied by exposing the same materials to 24 GeV/c proton radiation in ambient air, in inert gas at ambient temperature and in liquid helium.

## 2. Materials and Methods

### 2.1. The Samples

The different resin systems of this study are listed below:

- CTD101K; bisphenol-A (DGEBA) epoxy system with an anhydride hardener [21,22]; baseline impregnation of HL-LHC superconducting magnets [1];
- Polab Mix; four-component system with improved mechanical properties [23] through addition of a solvent-free, low viscous hot-curing polyglycol flexibiliser Araldite DY040 [24] to the CTD101K system; used for impregnation of superconducting [25] and resistive magnet coils [26];
- MSUT; three-component system (Araldite MY 740 (100 pbw)/Aradur HY 906 (90 pbw)/DY 062 (0.2 pbw)), used for impregnating of high-field magnets at the University of Twente [27];
- Araldite F; three-component system (Araldite F (100 pbw)/Aradur HY 905 (100 pbw)/flexibiliser DY 040 (10 pbw)) [28]; widely used in different resistive magnets like the QA magnets of the Large Electron Positron collider (LEP) and in superconducting magnets like the ITER PF coils. Araldite F properties after mixed gamma/neutron reactor irradiation are reported in [11];
- CTD425; epoxy–cyanate ester blend that was developed for ITER TF coils [29,30];
- MY750; two-component system (bisphenol A resin Araldite MY750 (100 pbw)/polyamine hardener Aradur HY5922 (55 pbw)) [31];
- Mix61; four-component system with amine-based hardener [32];
- Stycast 2850FT-23LV; charged epoxy system with amine-based hardener [33,34];
- Damisol 3418; one-component class H high-voltage solvent-free DGEBA epoxy–anhydride hardener system [35];
- Damisol 3420; one-component class F anhydride free epoxy system.

More information about these materials and their processing can be found in references [36,37,38,39].

### 2.2. Iradiations and Dosimetry

#### 2.2.1. Gamma Irradiation and Dosimetry

For gamma irradiations a ^60^Co source, emitting photons with energies of 1.17 MeV and 1.33 MeV (average: 1.25 MeV), has been used. Irradiations were performed at the Gammatec facility at the Marcoule site of the company Synergy Health Marseille SAS, Marseille, France (a Steris company) with a dose rate of about 2 kGy/h in ambient air at a temperature of 20–25 °C. During irradiation, the sample holders are continuously rotated to improve dose homogeneity.

Dosimetry measurements were performed at the beginning of the ^60^Co irradiation and after every 2 MGy dose step with dosimeters that were attached at different positions inside and outside the sample holders. Dosimetry measurements were done either by measuring the radiation-induced changes of the optical density of red Perspex 4043 (PMMA) or by alanine loose-pellet dosimeters [40].

#### 2.2.2. Proton Irradiation and Dosimetry

Proton irradiations have been achieved at the CERN IRRAD facility with a pulsed 24 GeV/c proton beam from the CERN PS accelerator, delivering about 7 × 10^11^ protons per pulse of ~400 ms length. The average proton fluence of about 1.4 × 10^16^ p/cm^2^ per week corresponds to a dose of about 4 MGy per week in samples of 10 mm × 10 mm cross section. During one year of operation at IRRAD, the total dose reachable depends both on the length of the run as well as on the facility occupancy (e.g., time which is possible to allocate to a given experiment w.r.t. the overall user requests). Typically, yearly doses in the range 60–100 MGy can be accumulated at IRRAD for experiments performed at RT.

The proton fluence is determined by measuring the activation of thin aluminium witness foils that are irradiated simultaneously with the samples to be tested. The result is the average fluence in a 10 mm × 10 mm Al foil cross section. The accuracy of these fluence measurements is ±7% [41].

**Figure 2:**
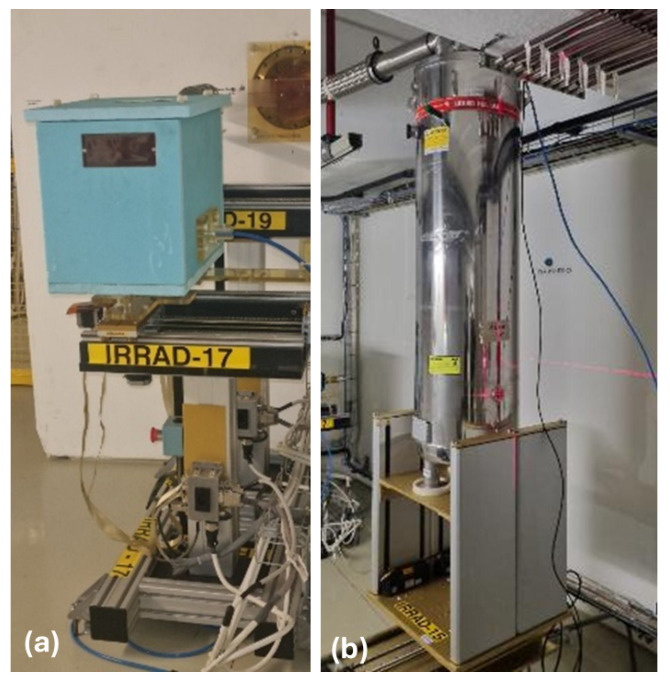
(**a**) Box for inert gas irradiation and (**b**) liquid helium cryostat in IRRAD 24 GeV/c proton irradiation facility.

For inert gas irradiations, at IRRAD the samples are inserted in a box made of Styrofoam brand extruded polystyrene (Figure 2a). During the entire irradiation duration a continuous nitrogen flow of 200 L/h at ambient temperature is maintained. Prior to the irradiations, the oxygen content in the inert gas was controlled with a Metrotec portable GSM touch zirconium dioxide oxygen analyser [42].

The cold irradiation results presented in this article have been achieved in liquid helium (LHe), using a cryostat installed in IRRAD (Figure 2b). The cryostat needs to be refilled daily with LHe from outside of the IRRAD facility via a flexible helium transfer line. During irradiation the cryostat material is activated and several weeks of decay are required prior to its opening to access the samples. These reasons limit irradiations in the LHe cryostat to few weeks (typically three) and towards the end of the irradiation run only. With the LHe cryostat, three dose steps at 4.2 K (3 MGy, 5 MGy and 10 MGy) could be achieved during three successive IRRAD operation years.

#### 2.2.3. Mixed Neutron/Gamma Irradiation at the TRIGA Mark II Reactor

Mixed gamma/neutron irradiations at the central sample position of the TRIGA Mark II reactor at the University of Vienna have been performed with an approximate gamma dose rate of 600 kGy/h, a fast neutron flux density *Φ_f_* = 3.5 × 10^16^ m^−2^ s^−1^ (E > 0.1 MeV), and a total neutron flux density of *Φ_f_* = 1.3 × 10^17^ m^−2^s^−1^, respectively [43]. In a typical epoxy resin, a fast neutron fluence of 1 × 10^22^ m^−2^s^−1^ (E > 0.1 MeV) corresponds to a dose of about 70 MGy (49 MGy from gamma rays and 21 MGy from neutrons). The combined neutron and gamma dose rate is 860 kGy/h.

Before irradiation, the samples were sealed in quartz glass capsules that were either evacuated to a pressure below 5 × 10^−2^ mbar and then sealed (1, 3 and 10 MGy dose steps), or the quartz glass capsules were filled with ambient air (1, 3, 20, 40, 60 MGy dose steps). Vacuum sealed capsules enabled us to measure the irradiation-induced gas production as described in [20].

Irradiations to 30, 50 and 70 MGy have been achieved in two dose steps, one step to 10 MGy encapsulated in vacuum, and a subsequent dose step to either 20, 40 or 60 MGy with the samples encapsulated in ambient air. The quartz capsules were immersed in the reactor pool water, which has an approximate temperature of 50 °C. A sample temperature rise to about 70 °C during irradiation is estimated. The highest dose step of 120 MGy was reached by immersing samples directly in the reactor pool water.

#### 2.2.4. Mixed Neutron/Gamma Irradiation at the n_TOF Spallation Source

The CERN n_TOF facility [44] is a neutron spallation source. Material irradiations can be performed in parasitic mode at the NEAR n_TOF irradiation station [45]. Rabbit tubes enable sample exchange without opening the shielding around the n_TOF target. Unless explicitly mentioned, all irradiations reported here were performed at the Rabbit 2 position.

Pulses of about 7 × 10^12^ protons from the CERN PS accelerator are delivered onto the n_TOF target. The neutron fluences and the simultaneous gamma doses per proton pulse at the different n_TOF irradiation positions have been determined by Monte Carlo simulations [46]. The dose that is absorbed under a certain neutron fluence depends on the neutron nuclear cross section of the elements present in the sample material. At the Rabbit 2 position, in a typical epoxy resin (composed of H-47.5 at.%, C-47.0 at.%, O-7.5 at.%), 0.6 Gy are absorbed per proton pulse on target. In total, 75% of this dose is due to neutrons.

Irradiations have been performed in ambient air at a temperature between 20 and 25 °C. A dose of about 2.5 MGy is accumulated during a one-year operation of n_TOF.

### 2.3. Effect of the Elemental Composition on the Dose Absorbed from the Different Radiation Sources

The energy absorbed from the different radiation sources depends on the elemental composition of the sample. To illustrate how far the dose values absorbed in the different samples deviate from each other, the dose absorbed in four epoxy systems has been calculated as a function of photon, proton and neutron energy. The uncertainties of the different dosimetry methods and the calculations are described in more detail in reference [41]. The assumed elemental compositions are summarised in Table 1.

#### 2.3.1. Effect of the Elemental Composition on the Photon Dose

**Figure 3:**
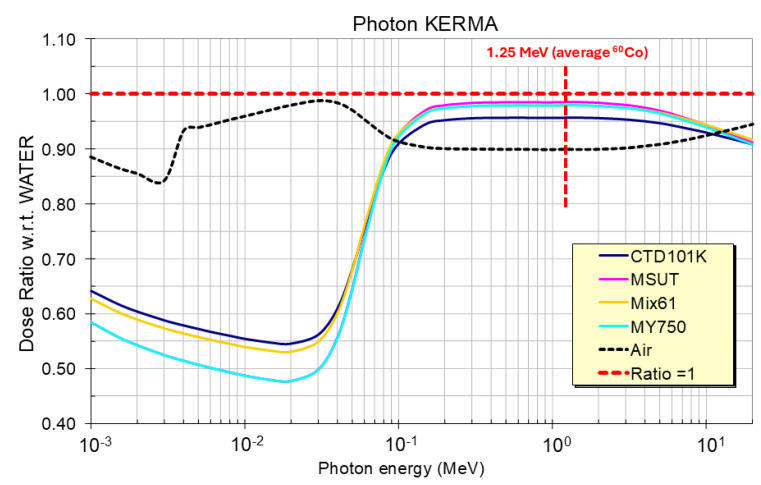
Photon dose ratio in four epoxy resin systems and in air versus photon dose in water for photon energies ranging from 1 keV to 20 MeV.

The ^60^Co dosimetry results reported here have been determined for water. Therefore, the ratio of energy absorption coefficients of epoxy to water are plotted in Figure 3 as a function of the photon energy [47]. When exposed to the same photon fluence, at a photon energy of 1.25 MeV the four epoxy systems absorb between 2% to 4% less dose than water (Table 1). In the following this small difference is neglected, and water equivalent ^60^Co gamma ray dose values are used for all polymers of the present study.

#### 2.3.2. Effect of the Elemental Composition on the Proton Dose

**Figure 4:**
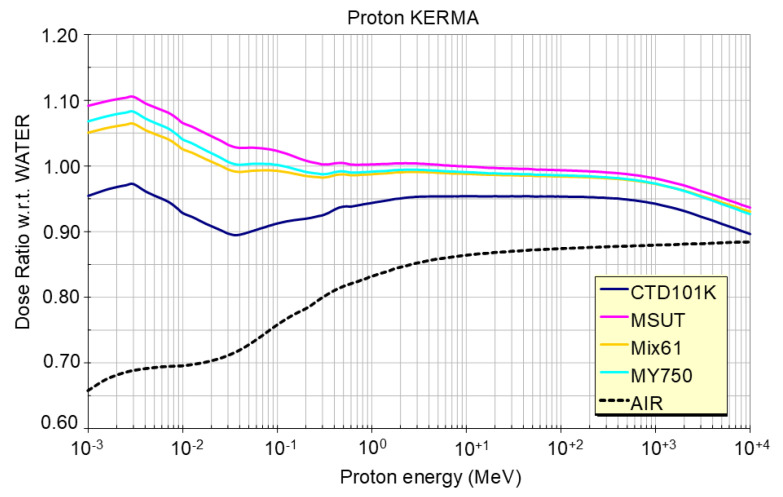
Proton dose ratio in four epoxy resin systems and in air versus water for proton energies ranging from 1 keV to 10 GeV. The 10 GeV value can be considered as representative of the one for 24 GeV/c (~23 GeV) protons.

The proton energy loss ratios in epoxy versus water are plotted in Figure 4 as a function of the proton energy. In the proton energy range from 1 keV to 1 GeV, deviations of the epoxy resin dose from the proton dose absorbed in water are within ~5%. In the 10s of GeV region, this slightly increases but still remains well below 10%. Differences in the dose absorbed in the different epoxy resins are <±5%. Throughout this article, for all polymers, the same 24 GeV/c proton fluence-to-dose conversion is performed, assuming the dE/dx for a typical epoxy resin equal to 1.87 MeV × cm^2^/g [47].

#### 2.3.3. Effect of the Elemental Composition on the Neutrom Dose

**Figure 5:**
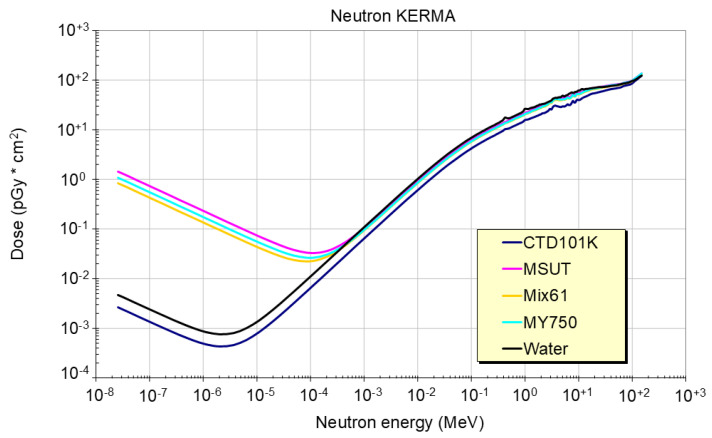
Neutron energy loss in four epoxy resin systems and in water for neutron energies ranging from 0.1 eV to 10 GeV.

Energy spectra of neutrons from fission reactors or spallation sources cover an energy range of several orders of magnitude (Figure 5). For thermal and epi-thermal neutrons (0.1–1 keV), the absorbed dose of the epoxy resins is strongly influenced by the nitrogen content. Above that energy range, the proton recoils due to the presence of hydrogen prevailing. In the fast neutron energy range (>0.1 MeV), the dose absorbed in these epoxy resins is about 20% lower than that absorbed in water. The dose absorbed by water and the epoxy resin from high energetic neutrons (>20 MeV) is nearly equal.

The energy deposited by the combined gamma/neutron irradiation in the TRIGA Mark II reactor has been calculated as outlined in [48]. In the TRIGA reactor radiation field, the dose rate is primarily determined by the hydrogen content of the epoxy resin. Table 1 compares the effect of the elemental resin composition on the dose absorbed with the different sources.

**Table 1:** Elemental composition of four epoxy resin systems and absorbed dose from ^60^Co gamma irradiation relative to water. For 24 GeV/c proton irradiation and mixed gamma/neutron irradiation in the TRIGA Mark II reactor, doses in the different epoxy systems are compared to the average epoxy dose.

	Composition in wt.%				Dose in % of H_2_O Dose	Dose in % of Average Epoxy Dose	
	C	H	O	N	^60^Co Gamma	24 GeV/c Proton	TRIGA
CTD101K	70.9	6.4	22.7	0	**96**	**97**	**92**
MSUT	64.9	9.5	7.4	18.2	**98**	**101**	**104**
Mix61	62.8	8.9	17.7	10.6	**96**	**101**	**102**
MY750	68.4	8.9	9.1	13.6	**98**	**101**	**102**
Water	0	11.2	88.8	0	**100**	**107**	**X**

### 2.4. Dynamic Mechanical Analysis (DMA)

DMA measurements in torsion mode were performed with a Dynamical Mechanical Analyser MCR702e from Anton Paar, Graz, Austria, using rectangular beams with a nominal sample thickness = 4 mm, width = 10 mm and length = 40 mm. The evolutions of the storage modulus (G′) and the loss modulus (G″) during temperature sweeps with a temperature ramp of 2 K/min were recorded at a frequency of 1 Hz.

In the following the glass transition temperature (*T*_g_) is defined as the temperature at the maximum of the G″ peak [49,50]. *G*′_rubbery_ is measured at the end of the temperature sweep when the sample is heated above its *T*_g_. For an ideal rubber [51,52,53,54], the density of (elastically effective) cross-links (*ν*) of a thermoset can be calculated according to (1) from *G*′_rubbery_, the gas constant (R = 8.314 J mol^−1^K^−1^) and the absolute temperature (T):

*ν* = *G*′_rubbery_/*R* × *T* (1)

The molecular weight between cross-links (*M_c_*) can be calculated according to (2) from ν and the polymer density (ρ):

*M_c_* = *ρ*/*ν* (2)

**Figure 6:**
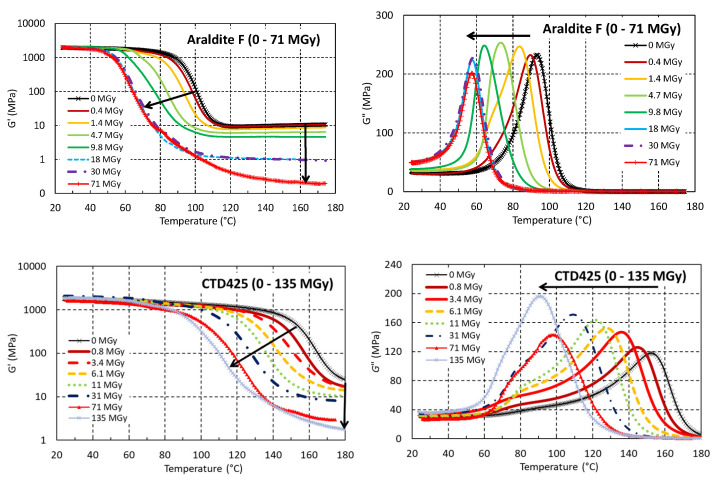

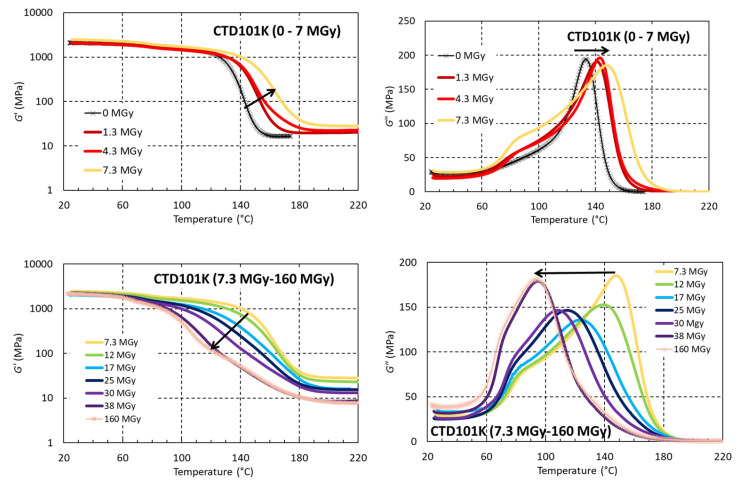
*G*′(T) and *G*″(T) of Araldite F, CTD425 and CTD101K before and after different 24 GeV/c proton doses absorbed in ambient air. The arrows indicate the shifts of *G*′(T) and *G*″(T) with increasing dose.

Dose-dependent changes of *T*_g_ and *G*′_rubbery_ have been recorded to monitor irradiation-induced cross-linking and chain scission. Unless explicitly stated, all *T*_g_ and *G*′_rubbery_ values reported in this article have been determined during the first temperature cycle following the irradiations. Figure 6 shows how the *G*′(T) and *G*″(T) curves of three resin systems shift with increasing dose. The Araldite F epoxy resin and the CTD425 epoxy–cyanate ester blend systems exhibit a continuous reduction in *T*_g_ and *G*′_rubbery_ with an increasing dose. For the CTD101K epoxy system, *T*_g_ and *G*′_rubbery_ initially increase due to irradiation-induced cross-linking. Doses > 7 MGy cause a continued reduction in *T*_g_ and *G*′_rubbery_ up to about 40 MGy. Further increasing the dose to 160 MGy changes the *G*′(T) and *G*″(T) only slightly.

### 2.5. Three-Point Bending Tests

Three-point bending tests were performed in short beam configuration according to ISO 14130:1997 [55]. Samples with 40 mm length, 10 mm width and with a nominal thickness of 4 mm were tested using Ø = 4 mm loading supports and a Ø = 10 mm bending die. The span length was 5× the nominal sample thickness, and the crosshead speed was 1 mm/min. The flexural stress was calculated according to Equation (1) from load (*F*), specimen width (*b*), specimen thickness (*h*), support span (*L*), and displacement (*s*):

(3) $$Flexural\;stress\;(MPa):\;\sigma \;=\;\;\frac{3\;\times \;F\;\times \;L}{\begin{array}{c} 2\;\times \;b\;\times \;h^{2} \end{array}}$$

## 3. Results

### 3.1. Dose Dependence of Mechanical Strength and Cross Link Density up to 30 MGy

**Figure 7:**
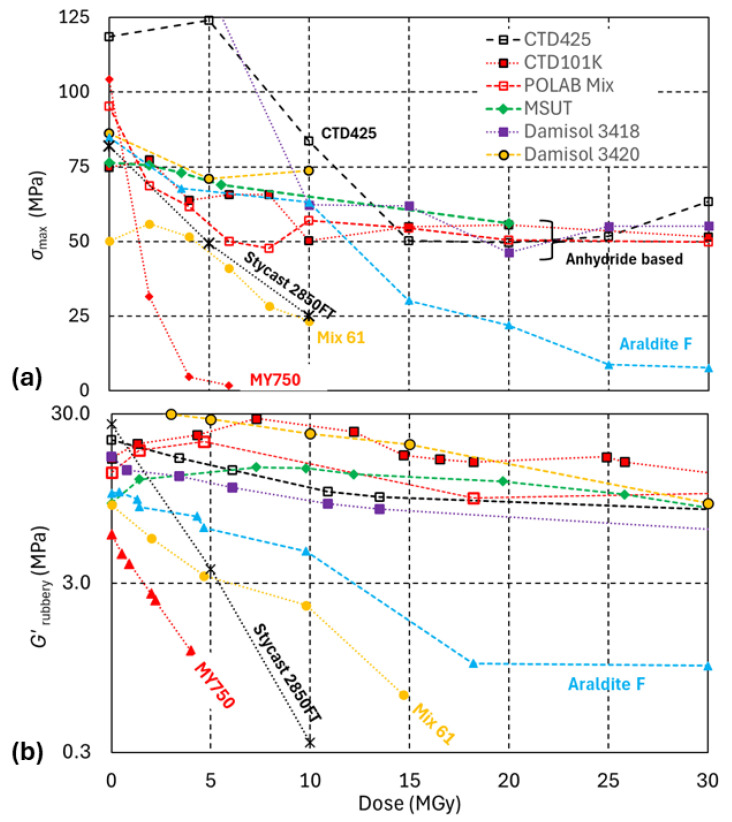
(**a**) *σ*_max_ and (**b**) *G*′_rubbery_ as a function of dose absorbed in ambient air up to 30 MGy. Changes in *σ*_max_ and *G*′_rubbery_ are measured after proton and after gamma irradiation, respectively.

Figure 7 compares the evolutions of the three-point bending stress at fracture (*σ*_max_) and of *G*′_rubbery_ of identical epoxy resin systems as a function of dose up to 30 MGy. Irradiation of mechanical test samples was performed with ^60^Co gamma rays with a dose rate of 2 kGy/h in ambient air. The non-destructive *G*′_rubbery_ measurements have been performed after proton irradiation in ambient air.

Similar trends are observed in the *G*′_rubbery_ and in the *σ*_max_ evolutions. A comparatively faster decrease in *σ*_max_ and *G*′_rubbery_ occurs for the epoxy systems with amine-based hardeners (MY750, Mix61, Stycast 2850). As revealed by *σ*_max_ and *G*′_rubbery_, the epoxy systems with anhydride-based hardeners and the epoxy–cyanate ester blend have outstanding radiation hardness and maintain a *σ*_max_ > 50 MPa up to at least 30 MGy. The Araldite F system with anhydride hardener blend Aradur HY 905 is less radiation-hard than the other anhydride hardener-based systems.

From the comparison above, it is concluded that monitoring dose-dependent variations in cross-link density derived from *T*_g_ and *G*′_rubbery_ is a meaningful basis for comparing the radiation hardness of epoxy resin systems in different radiation fields and irradiation environments.

### 3.2. Effect of Ambient Air Irradiation on Cross-Link Density up to 170 MGy

**Figure 8:**
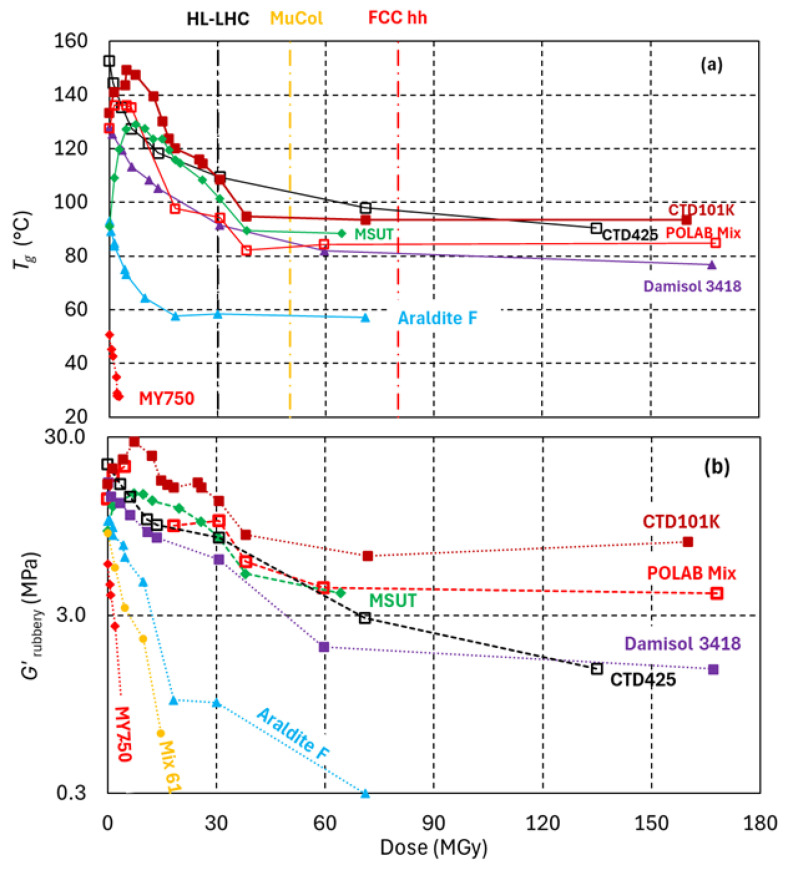
(**a**) *T*_g_ and (**b**) *G*′_rubbery_ of epoxy resin systems as a function of 24 GeV/c dose absorbed in ambient air. The vertical dashed lines indicate the dose limits assumed for HL-LHC, FCC hh and muon collider projects.

The *T*_g_ and *G*′_rubbery_ evolutions of epoxy resin systems under 24 GeV/c proton irradiation in ambient air are compared in Figure 8. The peak dose of 170 MGy has been achieved with 24 GeV/c protons in ambient air during two entire years of IRRAD operation. The evolutions of *T*_g_ and *G*′_rubbery_ are likewise influenced by cross-link density and follow similar trends.

The anhydride hardener-based epoxy resin systems referred to as MSUT, CTD101K and POLAB Mix exhibit a similar irradiation response, with an initial *T*_g_ increase due to irradiation-induced formation of new cross-links. After 6 to 10 MGy, a *T*_g_ peak is reached, and higher doses reduce *T*_g_. At about 40 MGy, a *T*_g_ plateau is reached. Further increasing the dose up to 170 MGy changes *T*_g_ only slightly.

The addition of the DY040 flexibiliser reduces *T*_g_ of the POLAB Mix by approximately 10 °C relative to the non-flexibilised CTD101K [23]. The *T*_g_ difference is maintained during the entire dose range that has been explored.

MSUT, CTD101K and POLAB Mix, Damisol 3418 and the CTD425 epoxy–cyanate ester systems maintain intermediate cross-link densities up to at least 170 MGy. These materials are expected to retain substantial mechanical strength after exposure to doses exceeding 100 MGy, which will be verified through mechanical testing after ongoing irradiations.

The least radiation-hard epoxy resin system of the present study is MY750, with an amine-based hardener. MY750 becomes unusable when doses exceed 2 MGy.

### 3.3. Effect of the Radiation Environment and Temperature

**Figure 9:**
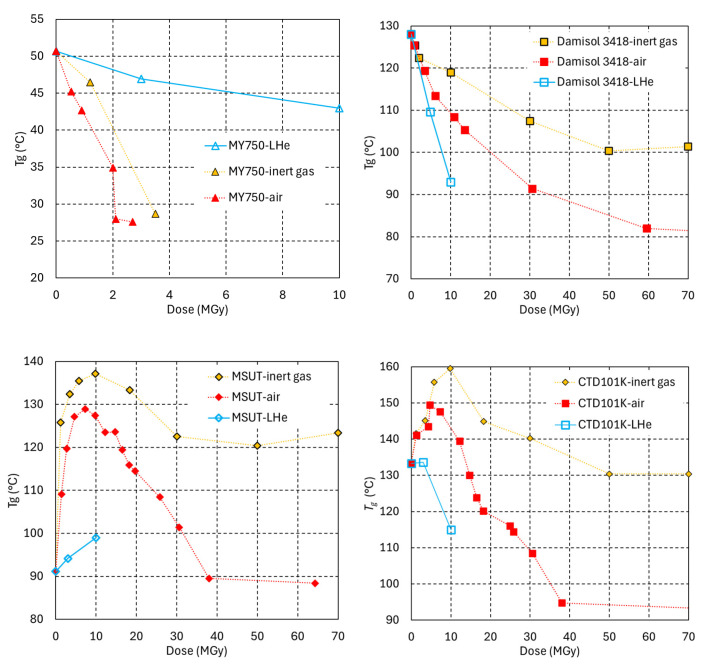
*T*_g_ as a function of the dose absorbed in ambient air, inert gas and in LHe.

To assess the influence of environmental oxygen and of the irradiation temperature, identical samples have been irradiated in ambient air, in inert gas at RT and immersed in LHe. All irradiations have been performed with the same proton source and dose rate. Figure 9 compares the *T*_g_ vs. dose dependences of different epoxy resin systems under irradiation in the different environments.

For all epoxies studied, irradiation-induced cross-linking in inert gas is enhanced with respect to the irradiation in ambient air, as manifested by a comparatively stronger initial *T*_g_ increase and higher *T*_g_ retained at high doses.

During irradiation in air, oxygen can diffuse into the sample and react with free radicals, thereby decreasing their probability of forming new cross-links. This effect is suppressed in an inert gas environment, leaving a greater number of free radicals available to participate in cross-linking reactions. It has been reported that the mechanical properties of CTD101K improve under inert gas irradiation [56], which may be explained by the comparatively higher cross-link density.

Cross-linking requires a certain degree of segmental mobility to enable reactive sites on different polymer chains to interact. Under irradiation at 4.2 K in LHe, segmental mobility is suppressed, and cross-linking is largely inhibited. This may at least partially explain the more rapid reduction in cross-link density observed in the CTD101K and Damisol 3418 systems.

The dose-dependent evolution of cross-link density is the result of simultaneous cross-linking and chain scission. For the MY750 system under LHe irradiation, a slower reduction in cross-link density is observed, suggesting that the chain scission yield is lower at 4.2 K than at RT, assuming that cross-linking cannot be enhanced at low temperature. For the other systems, the observed net changes in cross-link density result from the simultaneous cross-linking and chain scission, making it difficult to determine temperature-dependent variations in chain scission yields.

### 3.4. Effect of the Radiation Source

Identical epoxy resin systems have been characterised after irradiation with four different sources, notably 24 GeV/c protons, gamma rays from a ^60^Co source, by mixed neutron/gamma irradiation at the n_TOF spallation source, and by mixed gamma/neutron irradiation in the TRIGA fission reactor. The efficiency of the different sources in producing damage is compared based on dose-dependent changes in cross-link density, as revealed by DMA measurements. The evolutions of *T*_g_ and *G*′_rubbery_ under gamma and neutron-containing irradiations are compared with those under proton irradiation.

#### 3.4.1. Effect of ^60^Co Gamma Rays

**Figure 10:**
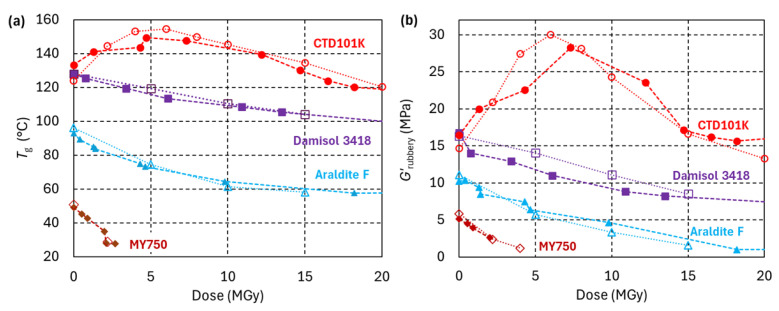
(**a**) *T*_g_ and (**b**) *G*′_rubbery_ evolution of different epoxy resin systems as a function of dose absorbed in ambient air. Full symbols represent proton irradiation and empty symbols, gamma irradiation.

Figure 10 compares the evolutions of *T*_g_ and *G*′_rubbery_ as a function of the absorbed dose under ^60^Co gamma and 24 GeV/c proton irradiation in ambient air. A certain dose absorbed from gamma rays and from protons has the same effect on the *T*_g_ and *G*′_rubbery_, suggesting that both radiation types have the same efficiency to produce radiation damage in epoxy resins.

#### 3.4.2. Effect of Neutron-Dominated Radiation at the n_TOF Spallation Source

**Figure 11:**
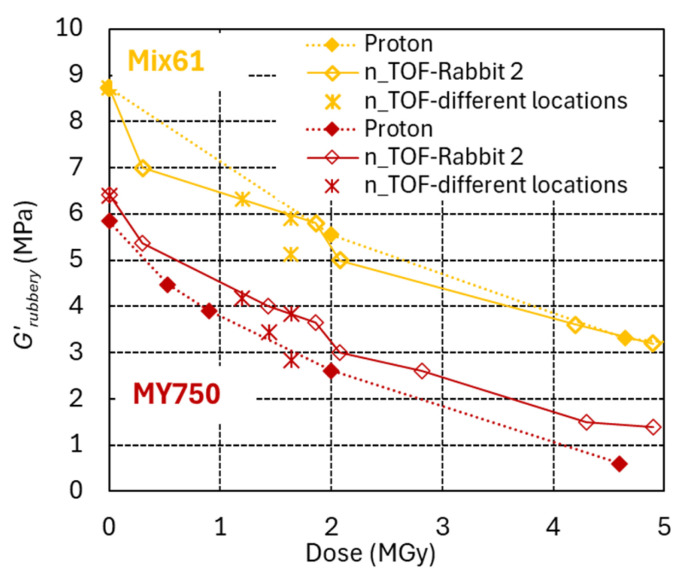
*G*′_rubbery_ of MY750 and Mix 61 versus dose absorbed in ambient air. Full symbols indicate proton irradiation. Empty symbols and asterisks denote neutron/gamma irradiation at NEAR n_TOF Rabbit-2, and at other NEAR n_TOF locations, respectively.

**Figure 12:**
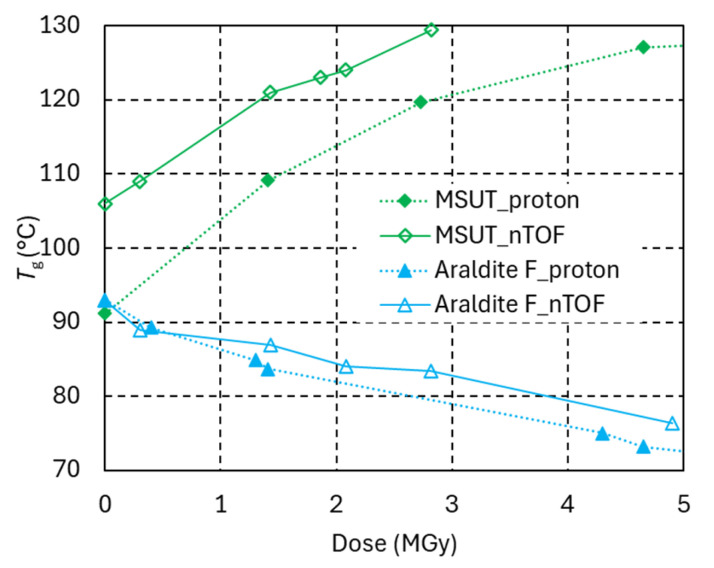
*T*_g_ of Araldite F and MSUT versus dose absorbed in ambient air. Full symbols indicate proton irradiation. Empty symbols denote neutron/gamma irradiation at NEAR n_TOF Rabbit-2.

The efficiency of neutron and proton irradiation to produce damage in epoxy resins with amine- and anhydride-based hardeners is compared in Figure 11 and Figure 12, respectively.

The highest dose achieved at NEAR n_TOF so far is approximately 5 MGy, accumulated over two years of operation. About 75% of this dose is caused by neutrons, and the remaining 25% is due to gamma rays.

The *G*′_rubbery_ versus dose evolutions of Figure 11 suggest that both radiation types have a similar efficiency with respect to producing chain scission in the MY750 and Mix61 epoxy systems with amine-based hardener. The scatter of the results suggests that the dose uncertainty in different n_TOF locations could be up to ±50%.

The *T*_g_ evolutions of the MSUT and Araldite F systems under the same radiation conditions are presented in Figure 12. Also, for these more-radiation-hard epoxy systems, the same trends are observed under both radiation types, suggesting that the same dose absorbed from neutrons and protons produces a similar damage in epoxy resins.

#### 3.4.3. Effect of Gamma/Neutron Radiation in the TRIGA Reactor

**Figure 13:**
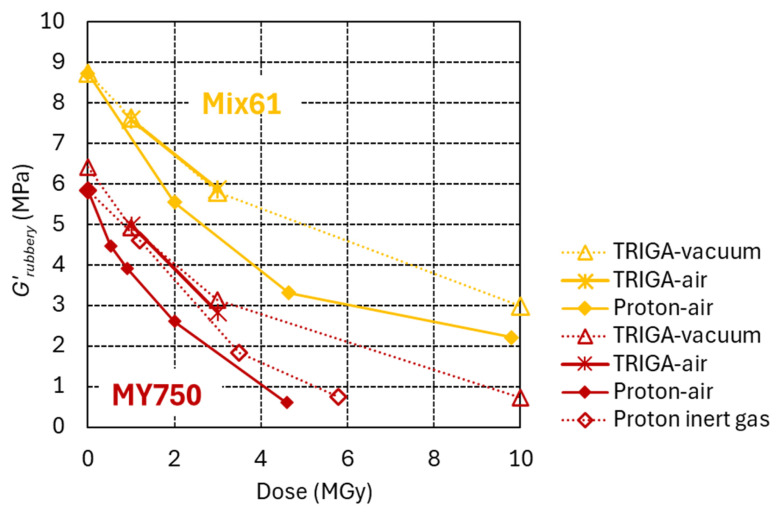
*G*′_rubbery_ of MY750 and Mix61 as a function of dose. Full and empty diamond symbols represent proton irradiation in air and in inert gas, respectively. In the TRIGA reactor, samples were encapsulated either in vacuum (open triangles, 1, 3 and 10 MGy) or in air (asterisks at 1 and 3 MGy).

The efficiency of reactor irradiation and proton irradiation to produce radiation damage in epoxy resins with amine-based hardeners is compared in Figure 13.

In the reactor, the samples were inserted into sealed quartz capsules, either in vacuum (1 MGy, 3 MGy and 10 MGy dose steps) or in air (1 MGy and 3 MGy dose steps). The *G*′_rubbery_ of MY750 and Mix61 after 1 MGy and 3 MGy is not affected by the presence of air in the capsules, presumably because oxygen diffusion is limited at the very high dose rate of about 900 kGy/h.

To reach the same *G*′_rubbery_, the dose required in the reactor is approximately twice that required for proton irradiation in air. As indicated by the comparison with proton irradiation of MY750 in inert gas, this difference can only partly be explained by the limited oxygen supply during reactor irradiation. Differences in the irradiation temperature may also contribute.

**Figure 14:**
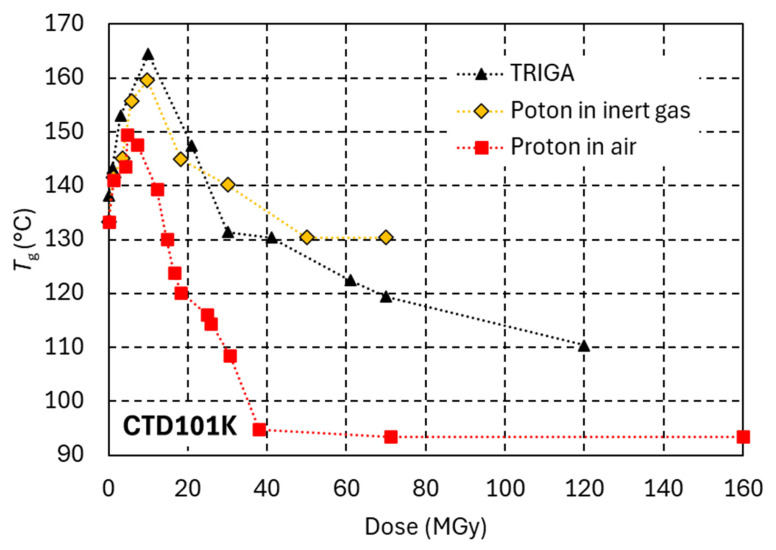
*T*_g_ of CTD101K as a function of dose under mixed gamma/neutron irradiation in the TRIGA reactor and under proton irradiation in ambient air and in inert gas.

Figure 14 compares the dose-dependent *T*_g_ evolutions of CTD101K under reactor irradiation, proton irradiation in ambient air and proton irradiation in inert gas. As compared to proton irradiation in ambient air, reactor irradiation causes more cross-linking and subsequently a comparatively slower decrease in *T*_g_. At doses above 30 MGy in the reactor, a *T*_g_ plateau and *G*′_rubbery_ of about 15 MPa are maintained, indicating that irradiation-induced chain scission is compensated by the formation of new cross-links. The cross-linking and chain scission behaviour observed during reactor irradiation is similar to that obtained during proton irradiation in an inert gas atmosphere.

### 3.5. Effect of Post-Irradiation Heating

**Figure 15:**
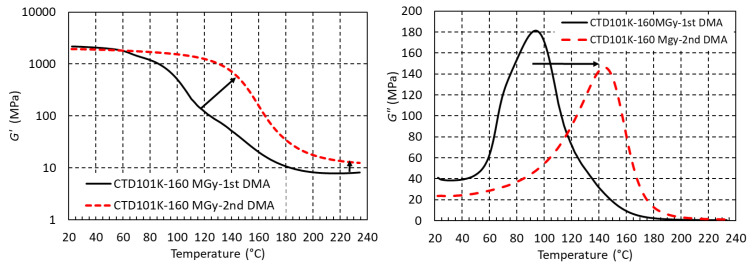
*G*′(T) and *G*″(T) of CTD101K exposed to 160 MGy measured during 1st and 2nd post-irradiation thermal cycle to 230 °C. The arrows indicate the shifts of *G*′(T) and *G*″(T) caused by post irradiation thermal curing.

During the DMA temperature sweeps, the samples are heated above *T*_g_. Free radicals, which have been produced during the prior irradiation ageing at RT, can become mobile and combine to form new cross-links. This can be seen in Figure 15, where thermal post-irradiation curing of the CTD101K system after 160 MGy irradiation in air leads to a 60% increase in *G*′_rubbery_ and a *T*_g_ increase of about 50 °C during the second DMA temperature cycle as compared to the first one.

**Figure 16:**
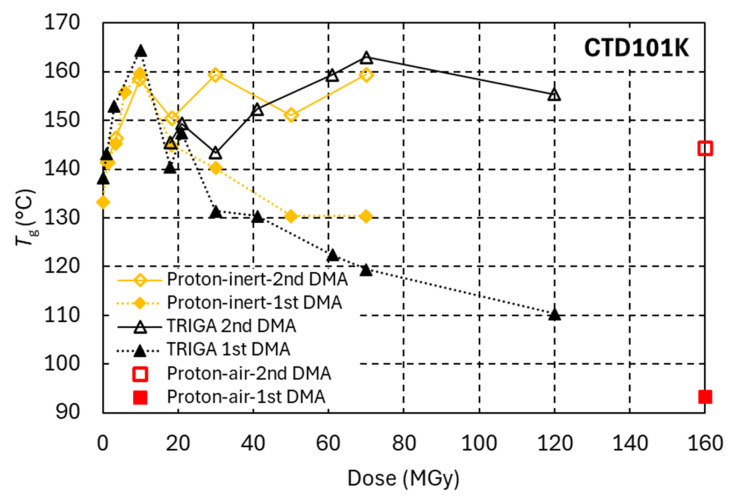
Effect of post-irradiation heating on *T*_g_ of CTD101K. Filled symbols indicate *T_g_* values measured during the first post-irradiation DMA temperature sweep at 2 K/min, while open symbols denote *T*_g_ measured during the second sweep following a first heat cycle up to 230 °C.

Figure 16 compares the CTD101K *T*_g_ versus dose evolution as measured during the first DMA temperature sweep and the subsequent second sweep. Thermal post-irradiation curing is comparatively small at low doses, but a progressively larger *T*_g_ increase is observed during the post-irradiation thermal cycle for doses exceeding 20 MGy.

## 4. Discussion

Different environmental and mechanical factors can act together and degrade polymer properties over time. In the present study we address radiation ageing, which is the dominating degradation mechanism in superconducting magnets. We have irradiated identical materials using four radiation sources: ^60^Co gamma rays, 24 GeV/c protons, and mixed neutron/gamma irradiation from a spallation source and in a fission reactor. Monitoring the dose-dependent relative changes of cross-link density, as manifested by changes in *T*_g_ and *G*′_rubbery_, allows for comparing the effects of these radiation types in varying radiation environments and in dependence of the irradiation temperature. Dose-dependent changes in the mechanical properties, as revealed by flexural tests, correlate with the changes of the cross-link density.

The comparison of the effect of neutron-dominated radiation, gamma and proton irradiation indicates that the energy that is absorbed (the total absorbed dose) is the primary parameter for the scaling of radiation effects in polymers. The primary radiation transfers its energy through the generation of secondary electrons, which have enough energy to break chemical bonds and to form radicals.

If irradiation is performed in the same environment, at the same temperature and with the same dose rate, a given dose absorbed from 24 GeV/c protons, ^60^Co gamma rays and neutron-dominated radiation from a spallation source produces similar damage in epoxy resin systems. The elemental composition influences the dose that is absorbed at a certain fluence, and in this way it influences the epoxy lifetime in a certain radiation field.

Because of the very high dose rates achievable, mixed gamma/neutron irradiation from fission reactors has been widely used to study radiation damage in radiation-hard polymers. Mixed gamma/neutron irradiation in the TRIGA Mark II reactor at a dose rate of about 900 kGy/h produces markedly different radiation effects compared with the slower irradiations in ambient air. The cross-link density evolves similarly during reactor and proton irradiation in inert gas, suggesting that limited oxygen diffusion is the main reason for this difference. The elevated sample temperature during reactor irradiation may also affect the cross-link density changes.

Gamma irradiation enables irradiation of large sample volumes, does not induce sample activation, and precise methods for the dose determination are available. This makes gamma rays well suited for irradiating samples for destructive mechanical testing. Under ambient air irradiation, epoxy resins with amine-based hardeners show severe mechanical degradation and become unusable at doses below 5 MGy. In contrast, radiation-hard systems with anhydride-based hardeners retain substantial mechanical strength up to at least 100 MGy [37]. The DMA results obtained after proton irradiation up to 170 MGy suggest that some epoxy systems may remain usable even at such high doses.

For the determination of the dose limit of polymer materials in accelerator magnets, the radiation conditions need to be considered. Organic insulation systems of resistive accelerator magnets are irradiated in ambient air at elevated temperature, typically at about 50 °C. In such an environment, the dose rate influences the dose limit [13,57]. In contrast, irradiation exposure of superconducting magnet insulation systems occurs at cryogenic temperatures in the absence of environmental oxygen.

Different results have been reported regarding the effect of irradiation temperature. Some studies indicate that irradiation at cryogenic temperatures produces damage comparable to that observed at ambient temperatures in radiation-hard epoxy systems and cyanate ester–epoxy blends [58,59,60]. A significant influence of irradiation temperature has been reported, for instance, in [61].

For the polymers included in this study, irradiation-induced cross-linking is almost completely suppressed when the irradiation occurs in liquid helium. The effect of the irradiation temperature on chain scission rates is material dependent, which might explain the contradicting observations that have been reported in literature. A focus of our future irradiation damage studies is the determination of the dose limits during irradiation under cryogenic conditions. To overcome the limitations of the LHe irradiation set-up, a cryocooler cooled set-up has been designed that will enable proton irradiations lasting several months per year in the IRRAD facility without helium consumption.

## 5. Conclusions

The total absorbed dose is the primary parameter for the scaling of radiation effects in polymers produced by different sources.

The irradiation environment and temperature can have a strong influence on radiation damage. At ambient temperature, irradiation-induced cross-linking is enhanced when oxygen diffusion into the sample is avoided. Irradiation-induced cross-linking in epoxy resins is strongly temperature-dependent, and it is almost completely suppressed during irradiation at 4.2 K. A strong temperature effect on irradiation-induced chain scission is observed for some epoxy resins.

Epoxy systems that exhibit irradiation-induced cross-linking maintain a higher cross-link density when irradiated in the TRIGA reactor. This is at least partly due to limited oxygen supply during irradiations with very high dose rate in the order of 1 MGy/h. Elevated sample temperature during reactor irradiation can also influence the resulting radiation damage.

The maximum dose to which epoxy resin systems can be exposed is strongly dependent on the hardener type, and it differs by two orders of magnitude. For the most-radiation-hard epoxy resin systems, beyond 40 MGy the evolution of radiation damage becomes very slow, at least up to 170 MGy.

## Data Availability

The original contributions presented in this study are included in the article. Further inquiries can be directed to the corresponding author.
